# Nomogram based on clinical features at a single outpatient visit to predict masked hypertension and masked uncontrolled hypertension: A study of diagnostic accuracy

**DOI:** 10.1097/MD.0000000000032144

**Published:** 2022-12-09

**Authors:** Hong Meng, Liang Guo, Bin Kong, Wei Shuai, He Huang

**Affiliations:** a Department of Cardiology, Renmin Hospital of Wuhan University, Wuhan, Hubei, PR China; b Cardiovascular Research Institute of Wuhan University, Wuhan, Hubei, PR China; c Hubei Key Laboratory of Cardiology, Wuhan, Hubei, PR China.

**Keywords:** masked hypertension, masked uncontrolled hypertension, nomogram, prediction model

## Abstract

Patients with masked hypertension (MH) and masked uncontrolled hypertension (MUCH) are easily overlooked, and both cause target organ damage. We propose a prediction model for MH and MUCH patients based on clinical features at a single outpatient visit. Data collection was planned before the index test and reference standard were after. Thus, we retrospectively collect analyzed 804 subjects who underwent ambulatory blood pressure monitoring (ABPM) at Renmin Hospital of Wuhan University. These patients were divided into normotension/controlled hypertension group (n = 121), MH/MUCH (n = 347), and sustained hypertension (SH)/sustained uncontrolled hypertension group (SUCH) (n = 302) for baseline characteristic analysis. Models were constructed by logistic regression, a nomogram was visualized, and internal validation by bootstrapping. All groups were performed according to the definition proposed by the Chinese Hypertension Association. Compared with normotension/controlled hypertension, patients with MH/MUCH had higher office blood pressure (BP) and were more likely to have poor liver and kidney function, metabolic disorder and myocardial damage. By analysis, [office systolic blood pressure (OSBP)] (P = .004) and [office diastolic blood pressure (ODBP)] (P = .007) were independent predictors of MH and MUCH. By logistic regression backward stepping method, office BP, body mass index (BMI), total cholesterol (Tch), high-density lipoprotein cholesterol (HDL-C), and left ventricular mass index are contained in this model [area under curve (AUC) = 0.755] and its mean absolute error is 0.015. Therefore, the prediction model established by the clinical characteristics or relevant data obtained from a single outpatient clinic can accurately predict MH and MUCH.

## 1. Introduction

Cardiovascular diseases are the leading cause of death and account for an estimated 30% of deaths worldwide.^[1]^ Hypertension is an overarching risk factor for cardiovascular morbidity and mortality, and it is the most critical contributor to the burden of disease and the leading cause of mortality worldwide.^[2,3]^ Studies have shown that hypertension is responsible for almost 13% of all deaths.^[1,4,5]^ Based on the current international guidelines, ambulatory blood pressure monitoring (ABPM) is widely accepted as the gold standard for the diagnosis of hypertension.^[6–8]^

Masked hypertension (MH) and masked uncontrolled hypertension are used to define people with normal office blood pressure (BP) but hypertension out-of-office, which ABPM determines.^[9]^ MH refers to patients in the untreated or initial stage of treatment, and masked uncontrolled hypertension (MUCH) refers to patients with prior hypertension treatments.^[10,11]^ A large number of studies have shown that the risk of target organ damage and cardiovascular and cerebrovascular disease in patients with MH and MUCH is similar to that in patients with sustained hypertension (SH) or sustained uncontrolled hypertension (SUCH), and is significantly higher than that in patients with normotension (NH) and CH.^[12–15]^

However, when performing ABPM, the number of measurements is higher, and the measurements are also taken during sleep, so it can produce discomfort and affect the patient’s sleep, acceptance, and compliance. Moreover, few primary care providers screen for MH, and little is known about primary care providers’ awareness, knowledge, and attitudes toward MH.^[16]^ Then, MH and MUCH patients are always ignored because of normal office BP. In conclusion, the above reasons often limit the popularity of ABPM.

The purpose of this study is to identify MH/MUCH patients through the prediction model established by the clinical characteristics of a single outpatient and the data of examination results.

## 2. Methods

### 2.1. Population

Ethics Committee of Renmin Hospital of Wuhan University approved the study, and the study involves patient consent. Data collection was planned before the index test and reference standard were after. The patients who underwent ABPM in our hospital from September 1st, 2020 to December 31st, 2020 were selected retrospectively. The inclusion criteria included age over 18 years’ old and complete and accurate ambulatory BP data (more than 20 BP measurements during the day, more than 7 BP measurements at night, and more than 27 BP measurements throughout the day).^[6,17,18]^ The exclusion criteria include secondary hypertension, life expectancy less than or equal to 12 months, serious diseases (such as acute myocardial infarction, acute renal failure requiring renal replacement therapy, and end-stage malignant tumors), mental diseases, inability to cooperate with ABPM, and other diseases leading to inaccurate BP measurement.^[17,19,20]^

### 2.2. Definition

The diagnostic criteria of hypertension proposed in this study are implemented according to the diagnostic criteria of Europe and China, that is, the diagnostic threshold of hypertension is 140/90 mm Hg.^[20–22]^ (Refer to Supplementary File 1a and Supplementary File 1b, Supplemental Digital Content 1, http://links.lww.com/MD/I59 for specific diagnostic criteria and hypertension subtypes.)

### 2.3. Measurement of BP in the office

The clinical BP was recorded by trained nursing staff in 3 separate visits on 1 day. During each measurement, use the verified upper arm electronic sphygmomanometer with a standard cuff to read the sitting BP twice, with an interval of 2 min. Rest quietly for 5 min before measurement, and record the average value of the two BP readings as the measured value of this BP. The mean value of three BP measurements was taken as the study’s consulting room BP value.^[6,19,23]^

### 2.4. Measurement of ambulatory BP

Participants completed 24-h ABPM using an ABPM instrument with an appropriately sized cuff in the arm where their BP measurements were high. Ambulatory BP monitors were read at least once per hour and at least 27 times throughout the day (at least 20 times during the day and 7 times during the night). ABPM was uniformly divided into a whole day, daytime (8:00‐22:00), and night periods (22:00‐8:00).^[6,24]^

### 2.5. Measurement of observation index

All tests were obtained as fasting in the early morning before the patient underwent ABPM. The collected parameters included the following: liver function (alanine aminotransferase (ALT), aspartate aminotransferase (AST)), lipid profiles (total cholesterol (Tch), triglycerides (TG), high-density lipoprotein cholesterol (HDL-C), low-density lipoprotein cholesterol (LDL-C), small dense low-density lipoprotein cholesterol (sdLDL-C), arteriosclerosis index (AI, TG/HDL-C-1)), renal function (uric acid (UA), urea, creatinine (Cr), estimated glomerular filtration rate (eGFR)), fasting plasma glucose (Glu), electrocardiographic indicators (electric axis, QRS duration, PR interval, QT interval, QTc, SV1, RV5, and RS)), Echocardiographic parameters (diameter of ascending aorta (AAOD), left atrial diameter (LAD), left ventricular end diastolic diameter (LVDD), right atrial diameter (RAD), right ventricular end diastolic diameter (RVDD), end diastolic interventricular septal thickness (IVSD), left ventricular posterior wall thickness at end diastole (LVPWD), left ventricular ejection fraction (LVEF), left ventricular mass index (LVMI)).

### 2.6. Statistical analysis

Because this study had partially missing data, imputation of missing values took the method of multiple imputations five times, and the numerical value of these imputations was taken as the final data of the study while missing categorical variables took the method of deletion and were excluded from this study.^[25]^ Statistical analysis was performed by R 4.1.3 and SPSS 27.0 in this study. Quantitative variables are expressed as mean ± SD (x ± s), and categorical variables are expressed as percentages (%). Continuous parametric data were compared using an unpaired Student *t* test. Non-parametric data were compared using the Mann–Whitney *U* test. Categorical variables were analyzed using the Chi-square test or Fisher exact test. Spearman rank correlation coefficients were calculated between candidate variables. Statistical significance was inferred at a two-sided *P* value < .05.

Univariate logistic regression analysis was used to find out the clinical characteristics and relevant data that have a potential relationship with MH. The clinical features strongly correlated with MH were found by multivariate logistic regression analysis. The model is simplified and the error caused by the overfitting of the model is prevented by the backward step method of logistic regression.

The risk prediction model is visualized by transforming the regression coefficient into the Nomo coefficient. According to the degree of influence of each variable on the model, the corresponding score is given, and the score of each patient is calculated after adding all the scores, to determine the probability of disease of the patient.^[26]^

Area under curve (AUC) value, sensitivity, specificity, and a cutoff value of the model were calculated by receiver operating characteristic (ROC) analysis. A new sample is established by repeated return sampling, and the model is tested with the new sample.^[27]^

## 3. Result

### 3.1. Clinical characteristics of hypertension subtypes

Of all 804 patients, 121 were NH and CH, 343 were MH and MUCH, and 302 were SH and SUCH. No adverse events occurred when index tests or reference standards were performed. Among these 804 patients were 458 (57%) males with an age interval distribution of 61 ± 0.5. In the subsequent subgroup analysis, we found that in terms of baseline characteristics, compared with patients with NH and CH, MH and MUCH patients were younger, had higher office BP, and presented with poorer metabolic profiles, including higher body mass index (BMI) and serum TG levels. Meanwhile, MH showed that the structure of the left ventricle was more abnormal. And the difference between MH/MUCH and SH/SUCH is the same as the difference between NH/CH and MH/MUCH. However, SH/SUCH patients presented with medication nonadherence, and more of them did not take any antihypertensive drugs (Table 1).

**Table 1:** Baseline clinical characteristics of patients.

	NH/CH (group 1)	MH/MUCH (group 2)	SH/SUCH (group 3)	*P* value (group 1–2)	*P* value (group 2–3)
Age, yr	64.24 ± 1.15	61.14 ± 0.74	59.33 ± 0.87	.008	.139
Male, %	47 (39%)	210 (61%)	187 (62%)	<.001	.7
OSBP, mm Hg	123.45 ± 0.85	128.61 ± 0.45	151.06 ± 0.65	<.001	NA
ODBP, mm Hg	69.1 ± 0.7	74.67 ± 0.45	86.43 ± 0.68	<.001	NA
OHR, bpm	78.13 ± 1.13	79.73 ± 0.82	83.72 ± 0.9	.464	.001
Hypertension duration, yr	8.31 ± 1.01	7.79 ± 0.57	8.33 ± 0.61	.957	.415
No. of antihypertensive drugs				.116	.007
0	45 (37%)	111 (32%)	68 (23%)		
1	24 (20%)	68 (20%)	60 (20%)		
2	31 (26%)	83 (24%)	78 (26%)		
3	17 (14%)	54 (16%)	68 (23%)		
4	4 (3.3%)	26 (7.5%)	23 (7.6%)		
5	0 (0%)	5 (1.4%)	5 (1.7%)		
Previous history					
AF, %	11 (9.1)	28 (8.1)	12 (4)	.7	.03
CHD, %	60 (50)	143 (41)	98 (32)	.11	.021
Stroke, %	27 (22)	84 (24)	83 (27)	.7	.3
CKD, %	4 (3.3)	21 (6.1)	26 (8.6)	.2	.2
TDM, %	22 (18)	91 (26)	74 (25)	.075	.6
Hyperlipidemia, %	28 (23)	112 (32)	96 (32)	.059	.9
BMI	22.77 ± 0.36	25.05 ± 0.25	24.97 ± 0.25	<.001	.921
Laboratory tests					
ALT	19.22 ± 0.96	23.48 ± 0.88	24.12 ± 0.99	.014	.809
AST	21.51 ± 0.65	22.9 ± 0.56	22.93 ± 0.55	.237	.952
ALT/AST	0.88 ± 0.03	0.99 ± 0.02	1.01 ± 0.02	.007	.618
Urea	5.49 ± 0.18	5.79 ± 0.11	6.16 ± 0.16	.057	.444
Cr	64.5 ± 1.46	72.98 ± 1.58	87.49 ± 5.66	.003	.143
Urea/Cr	0.09 ± 0	0.08 ± 0	0.08 ± 0	.285	.339
UA	337.74 ± 10.16	372.81 ± 5.59	395.61 ± 5.78	.001	.001
eGFR	89.69 ± 1.62	89.88 ± 1.07	88.61 ± 1.52	.726	.557
Glu	5.07 ± 0.1	5.55 ± 0.1	5.76 ± 0.11	.005	.104
K	4.02 ± 0.03	4 ± 0.02	5.48 ± 1.43	.416	.161
Na	142.43 ± 0.38	143.06 ± 0.19	142.67 ± 0.18	.358	.168
Cl	107.32 ± 0.46	107.51 ± 0.17	107.15 ± 0.17	.514	.065
Ca	2.24 ± 0.01	2.23 ± 0.01	2.25 ± 0.01	.951	.063
Tch	4.16 ± 0.09	4.2 ± 0.06	4.45 ± 0.06	.596	.003
TG	1.38 ± 0.08	1.8 ± 0.07	1.89 ± 0.07	.001	.051
HDL-C	1.18 ± 0.03	1.02 ± 0.01	1.07 ± 0.02	<.001	.067
LDL-C	2.32 ± 0.08	2.37 ± 0.04	2.51 ± 0.05	.41	.044
sdLDL-C	0.67 ± 0.03	0.75 ± 0.02	0.83 ± 0.02	.018	.011
AI	2.71 ± 0.1	3.25 ± 0.08	3.33 ± 0.07	<.001	.211
Echocardiography					
AAOD	32.08 ± 0.4	33.18 ± 0.2	33.45 ± 0.22	.003	.39
LVEF, %	58.45 ± 0.49	58.55 ± 0.23	58.91 ± 0.19	.551	.734
LVMI	83.65 ± 1.95	90.14 ± 1.2	94.13 ± 1.44	.01	.021
LVH, %	21 (17)	71 (20)	73 (24)	.5	.3
Electrocardiogram					
Electric axis	33.99 ± 4.09	28.8 ± 1.99	31.4 ± 2.1	.121	.283
QRS duration	97.4 ± 1.43	101.39 ± 0.93	102.21 ± 0.91	.001	.319
PR interval	158.97 ± 2.55	162.06 ± 1.43	162.93 ± 1.59	.19	.943
QT interval	391.53 ± 3.37	387.31 ± 1.97	383.3 ± 2.1	.261	.138
QTC	427.33 ± 2.76	426.67 ± 1.5	429.47 ± 1.52	.928	.083
SV1	0.69 ± 0.06	0.71 ± 0.03	0.81 ± 0.03	.329	.001
RV5	1.35 ± 0.05	1.39 ± 0.03	1.51 ± 0.04	.455	.017
RS	2.05 ± 0.08	2.1 ± 0.04	2.32 ± 0.05	.368	.001

AAOD = diameter of ascending aorta, AF = atrial fibrillation, AI = atherosclerosis index, ALT = alanine aminotransferase, AST = aspartate aminotransferase, BMI = body mass index, CH = controlled hypertension, CHD = coronary heart disease, CKD = chronic kidney disease, Cr = creatinine, eGFR = estimated glomerular filtration rate, Glu = glucose, HDL-C = high-density lipoprotein cholesterol, LDL-C = low-density lipoprotein cholesterol, LVEF = left ventricular ejection fraction, LVH = left ventricular hypertrophy, LVMI = left ventricular mass index, MH = masked hypertension, MUCH = masked uncontrolled hypertension, NH = normotension, ODBP = office diastolic blood pressure, OHR = office heart rate, OSBP = office systolic blood pressure, sdLDL-C = small dense low-density lipoprotein cholesterol, SH = sustained hypertension, SUCH = sustained uncontrolled hypertension, Tch = total cholesterol, TG = triglycerides, TDM = diabetes mellitus, UA = uric acid.

### 3.2. Results of univariate and multivariate logistic regression

In the univariate logistic regression analysis, gender, age, office BP, BMI, ALT, ALT/AST, Cr, UA, Glu, HDL-C, AI, AAOD, LVMI, and QRS duration were associated with the presence of MH and MUCH. In the multivariate logistic regression analysis, the office BP was significantly associated with the presence of MH and MUCH (Table 2).

**Table 2:** Logistic regression results.

Dependent: type	OR	95% CI (univariable)	*P* value (univariable)	OR	95% CI (multivariable)	*P* value (multivariable)
Female	0.41	0.27‐0.63	<.001	0.68	0.38‐1.21	.191
Age	0.98	0.97‐1.00	.032	1	0.97‐1.02	.718
OSBP	1.06	1.04‐1.09	<.001	1.04	1.01‐1.07	.004
ODBP	1.08	1.06‐1.11	<.001	1.05	1.01‐1.09	.007
BMI	1.14	1.08‐1.20	<.001	1.06	1.00‐1.14	.073
ALT	1.02	1.01‐1.04	.01	1.02	0.95‐1.12	.592
ALT/AST	2.64	1.41‐5.13	.003	0.55	0.06‐4.62	.594
Cr	1.02	1.01‐1.03	.002	1.01	0.99‐1.03	.385
UA	1	1.00‐1.01	.002	1	1.00‐1.00	.529
Glucose	1.26	1.08‐1.52	.008	1.14	0.93‐1.42	.224
TG	1.47	1.17‐1.92	.002	1.41	0.86‐2.47	.203
HDL-C	0.14	0.06‐0.29	<.001	0.03	0.00‐1.05	.06
AI	1.59	1.28‐1.98	<.001	0.59	0.20‐1.67	.321
AAOD	1.08	1.02‐1.14	.01	1.02	0.95‐1.10	.537
LVMI	1.01	1.00‐1.02	.016	1.01	1.00‐1.03	.084
QRS duration	1.02	1.00‐1.03	.027	1.01	0.99‐1.02	.325

AAOD = diameter of ascending aorta, AI = atherosclerosis index, ALT = alanine aminotransferase, AST = aspartate aminotransferase, BMI = body mass index, Cr = creatinine, CI = confidence interval, HDL-C = high-density lipoprotein cholesterol, LVMI = left ventricular mass index, ODBP = office diastolic blood pressure, OSBP = office systolic blood pressure, OR = odds ratio, TG = triglycerides, UA = uric acid.

### 3.3. Predictive model construction and internal validation

**Figure 1.**
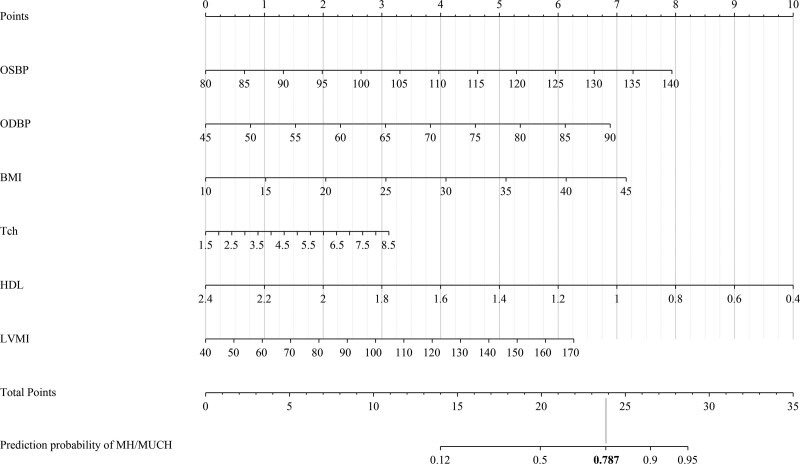
Nomogram of the scoring system predicting for MH/MUCH. The highest score of each index is 10 points. The best truncation value of the model is 0.787, and the corresponding score is about 24 points. MH = masked hypertension, MUCH = masked uncontrolled hypertension.

We developed a prediction model by using the predictors from the best fitting model obtained by logistic regression backward stepping method, which contains office BP, BMI, Tch, HDL-C, and LVMI. To visualize the system, we convert the regression results of each factor in the model into a Nomogram coefficient (nomogram coefficient = regression coefficient B* (maximum value of factor - minimum value of factor)), set the maximum score of the maximum factor of the coefficient as 10 points, and reduce the other factors in equal proportions (Supplementary File 2, Supplemental Digital Content 2, http://links.lww.com/MD/I60). Through the above principles and steps, we developed two scoring systems to predict MH and MUCH (Fig. 1). The maximum score of each item is set to 10 points, and the maximum total score is set to 35 points. The total score corresponds to the corresponding prediction probability.

**Figure 2.**
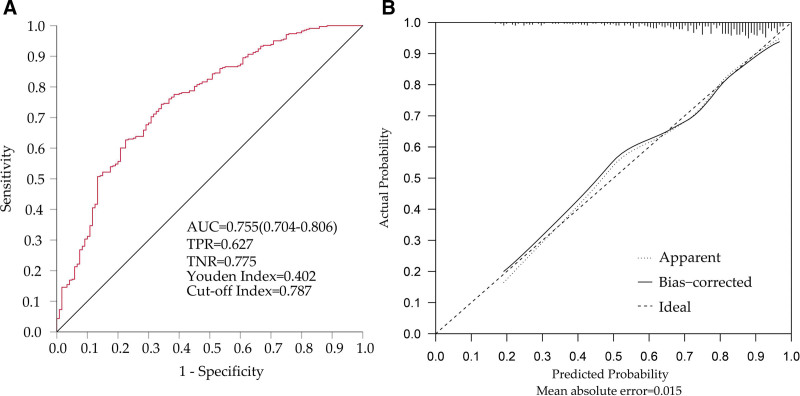
The results of testing prediction model accuracy. (a) ROC curve of the prediction model. The AUC of the ROC curve was 0.755, 95% CI: 0.704‐0.806, the TPR was 0.627, the (TNR) was 0.775, the Youden index was 0.402, and the optimal cutoff value was 0.787. (b) Internal validation using 10000 bootstraps resamples showed that only a small amount of the mean absolute error (0.015). AUC = area under curve, ROC = receiver operating characteristic, TPR = true positive rate, TNR = true negative rate.

The efficiency of the model was tested by the ROC curve. We found that the prediction efficiency of the model was positive (AUC = 0.755, 95% CI: 0.704‐0.806), and the sensitivity and specificity were within the acceptable range. Through ROC analysis, the cutoff value of our model is 0.787, and the score corresponding to the modified value on the nomogram is about 24 (Fig. 2a). The negative and positive cases obtained according to the gold standard ABPM and the cutoff value set by the diagnostic model can be seen in the Supplementary File 3, Supplemental Digital Content 3, http://links.lww.com/MD/I61. In addition, we internally validated the two sets of systems, and we found that both sets of models had good predictivity by the method of repeated sampling of the original sample 10000 times. As can be seen from the figure: from the figure, we can see that the apparent curve represented by the model constructed by the actual sample and the bias-corrected curve represented by the model of the new sample constructed by repeated sampling closely fit the ideal curve, which indicates that our model has good extrapolation (Fig. 2b).

## 4. Discussion

The main findings of this study are as follows: compared to NH and CH, MH and MUCH are associated with higher office BP and more disordered lipid metabolism; office BP is an independent risk factor for MH and MUCH; office BP, BMI, Tch, HDL-C, and LVMI are significantly association with MH and MUCH. The risk prediction model constructed by these clinical characteristics and indicators obtained from a single outpatient clinic can accurately predict MH and MUCH.

According to the definition of the guidelines, MH and MUCH are characterized by normotension in the office but hypertension out of the office. The only difference between them is whether they have a history of taking hypertension drugs. However, as stated in the introduction above: previous studies have shown that MH and MUCH are highly correlated with more severe target organ damage and worse prognosis. Although recent guidelines designate ABPM as the gold standard technique for diagnosing hypertension and distinguishing the types of hypertensions, it also has some limitations (such as affecting night sleep and high price). Therefore, it is unrealistic to conduct an ABPM examination for all populations. It is important to identify those at high risk of MH through the clinical characteristics related to a single outpatient visit.

Our study found that high office BP was highly correlated with MH and was an independent risk factor for MH. The results of the Spanish ambulatory BP registry and two other cohorts are also consistent with our findings.^[28,29]^

Our results also found that patients with MH and much showed worse metabolic status. These patients often have higher BMI, lower HDL-C, and higher serum Tch. Some previous studies have found MH to be more likely to be present in hyperlipidemia.^[30–32]^ This is also consistent with our findings. In addition, relevant studies have also confirmed that obesity or high BMI is highly correlated with MH and MUCH.^[33,34]^

In addition, our study also found that higher LVMI was associated with MH as well as much. Related studies on target organ damage of MH and MUCH found that MH and MUCH patients had higher LVMI.^[35,36]^ This is consistent with our findings. However, we evaluated the prevalence of left ventricular hypertrophy (LVH) among each group according to the LVH standard (LVMI ≥ 115 g/m^2^ in males and LVMI ≥ 95 g/m^2^ in females), but we found no significant statistical difference. This may be related to the selection of the population, the size of the sample, and the length of the course of hypertension.

In addition to the above indicators included in the model, our research results are worth discussing. Our study found no significant difference in the use of antihypertensive drugs between MH/ much patients and NH/ CH patients. This also indicates that much is not related to drug noncompliance. Relevant studies have also confirmed our view.^[37]^ In echocardiography, we found that the AAOD of MH/ MUCH patients was larger. Wang et al^[38]^ used a machine learning approach to perform automatic aorta measurement in thoracic CT images at nine key positions of 801 patients to predict MH. Better results were also finally obtained (AUC = 0.78). Therefore, this indicator may also be potentially related to MH and MUCH.

Whereas, in theory, a considerable proportion of studies found a strong association between CKD and MH.^[39,40]^ CKD and hypertension share similar risk factors and mutually contribute to each other. Still, our study did not find an association due to insufficient sample size and different study populations in our analysis. Our study only found that Cr was associated with MH and MUCH, and Cr in our study population was within the normal range. Furthermore, there is no relevant study evidence for a causal link between a history of chronic kidney disease (CKD) and MH.

In conclusion, our study provides a good evaluation model for predicting patients with hypertension outside the office. As far as we know, this is the first risk prediction model established by the relevant data obtained from a single outpatient service in China. By changing the scoring model, we can be sure to determine the patients who really should undergo ABPM. This can increase the patient’s medical experience and save medical costs.

## 5. Conclusion

Compared with NH/CH, patients with MH/MUCH had higher office BP and were more likely to have poor liver and kidney function, metabolic disorder and myocardial damage. The proposed model accurately predicts MH and MUCH based on the clinical features.

## 6. Limitation

This study is a retrospective study, and there will inevitably be a lack of relevant data. Although various statistical methods process these missing values, it is still difficult to avoid the relevant errors. Secondly, because the concept of occult hypertension is not popularized in China, most patients with normal BP in the clinic do not carry out ABPM in time, leading to a certain selection bias in the study. And this study only included the patient data for 4 months. Although the total sample number is more than 800, the sample number of this study is still small for the high prevalence of hypertension, which may also lead to some “no significant difference” results in this study. Therefore, future research in this area may include a larger sample size.

## Author contributions

**Conceptualization:** Bin Kong.

**Data curation:** Hong Meng.

**Formal analysis:** Liang Guo.

**Methodology:** Bin Kong.

**Project administration:** He Huang.

**Supervision:** He Huang.

**Software:** Liang Guo.

**Writing – original draft:** Hong Meng.

**Writing – review & editing:** He Huang, Wei Shuai.

## Supplementary Material


