# Supplementary material for: Nomogram based on clinical features at a single outpatient visit to predict masked hypertension and masked uncontrolled hypertension: A study of diagnostic accuracy
Source: Medicine (Baltimore). 2022 Dec 9;101(49):e32144. doi: 10.1097/MD.0000000000032144 (PMC9750695; doi:10.1097/MD.0000000000032144)
Supplement: Supplementary file 1 [file medi-101-e32144-s001.pdf]

Supplementary File 1a. Different types of hypertensions

Antihypertensive drugs	ABPM / Office/Clinic	Normal	Hypertension
No	Normal	Normotensive	Masked hypertension
	Hypertension	Whitecoat hypertension	Sustained hypertension
Yes	Normal	Controlled hypertension	Masked uncontrolled hypertension
	Hypertension	Whitecoat uncontrolled hypertension	Sustained uncontrolled hypertension

Supplementary File 1b. Comparison of American and European Society Definitions of Hypertension

		Systolic (mmHg)	Diastolic (mmHg)
Office/Clinic BP		140	90
ABPM	Daytime mean BP	135	85
	Nighttime mean BP	120	70
	24-hour mean BP	130	80
HBPM mean BP		135	85

ABPM: Ambulatory blood pressure monitoring; HBPM: Home blood pressure monitoring

BP: Blood pressure;
