# Supplementary material for: Nomogram based on clinical features at a single outpatient visit to predict masked hypertension and masked uncontrolled hypertension: A study of diagnostic accuracy
Source: Medicine (Baltimore). 2022 Dec 9;101(49):e32144. doi: 10.1097/MD.0000000000032144 (PMC9750695; doi:10.1097/MD.0000000000032144)
Supplement: Supplementary file 2 [file medi-101-e32144-s002.pdf]

Supplementary File 2. The transformation process from regression model to Nomogram.

factor	regression coefficient	The absolute value of regression coefficient	minimum value	Maximum value	Nomograph coefficient	Maximum point
OSBP	0.044	0.044	80	140	2.64	7.89
ODBP	0.051	0.051	45	90	2.295	6.85
BMI	0.068	0.068	10	45	2.38	7.11
Tch	0.149	0.149	1.5	8.5	1.043	3.12
HDL-C	-1.674	1.674	0.4	2.4	3.348	10
LVMI	0.016	0.016	40	170	2.08	6.21

Through logistic regression modeling, the regression coefficients of each factor in the model are obtained. The Nomograph coefficient is obtained by multiplying the regression coefficient by the range of each factor. Specifies that the factor with the largest Nomograph coefficient in the model corresponds to the largest score. The scores of other factors are reduced in proportion to the Nomograph coefficient. For example, in the NH-MH group, through calculation, the Nomo coefficient of HDL-C is the largest, so it is defined as 10 points. Then the Nomograph coefficient of ODBP is about one-third of TG, so the ODBP score is 3.12. OSBP: Office systolic blood pressure; ODBP: Office diastolic blood pressure; BMI: Body mass index; Tch: Total cholesterol; HDL-C: High-density lipoprotein cholesterol; LVMI: Left ventricular mass index.
