# Supplementary material for: Nomogram based on clinical features at a single outpatient visit to predict masked hypertension and masked uncontrolled hypertension: A study of diagnostic accuracy
Source: Medicine (Baltimore). 2022 Dec 9;101(49):e32144. doi: 10.1097/MD.0000000000032144 (PMC9750695; doi:10.1097/MD.0000000000032144)
Supplement: Supplementary file 3 [file medi-101-e32144-s003.pdf]

Supplementary File3. Cross tabulation of the index test results

Predict model	ABPM		Total
	Present	Absent	
Positive	215	27	242
Negative	128	93	221
Total	343	120	463(N)
